# Efficacy and Safety of Esmolol in Neonatal Cardiac Surgery with Cardiopulmonary Bypass (CPB) for d-Transposition of the Great Arteries (d-TGA)

**DOI:** 10.1007/s00246-024-03671-x

**Published:** 2024-10-09

**Authors:** Anna-Eva Blank, Thomas Zajonz, Inga Gruschwitz, Christoph Neuhäuser, Hakan Akintürk, Christian Jux, David Backhoff

**Affiliations:** 1 Pediatric Cardiology, Pediatric Heart Center, Justus-Liebig University Giessen, Giessen, Germany; 2 Pediatric Anesthesiology, Pediatric Heart Center, Justus-Liebig University Giessen, Giessen, Germany; 3 Pediatric Cardiac Surgery, Pediatric Heart Center, Justus-Liebig University Giessen, Giessen, Germany; 4 Department of Pediatric Cardiology, Intensive Care Medicine and Congential Heart Disease, Pediatric Heart Center, Justus-Liebig University Giessen, Feulgenstr. 10–12, 35392 Giessen, Germany

**Keywords:** Esmolol, Beta-blocker, Transposition of the great arteries, Congenital heart disease, Neonatal intensive care, Neonatal cardiac surgery

## Abstract

**Objective:**

D-Transposition of the great arteries (d-TGA) is the most common congenital heart disease requiring surgical correction within the neonatal period. Sinus tachycardia often persists postoperatively, potentially affecting cardiac function. This study aimed to investigate the efficacy and safety of the short-acting beta-1-selective beta-blocker esmolol in controlling heart rate in neonatal cardiac surgery with cardiopulmonary bypass (CPB).

**Methods:**

A retrospective cohort study was conducted on neonates undergoing surgery for d-TGA. The study cohort included 112 patients, divided into an esmolol intervention group (*n* = 57) and a control group (*n* = 55). Baseline characteristics, hemodynamic parameters and outcome measures were assessed.

**Results:**

In the esmolol group, median heart rate at ICU admission was significantly higher compared to the control group (155 vs. 147 bpm, *p* = 0.018). After a median time of 11 h, heart rate was lower among the esmolol patients (135 vs. 144 bpm, *p* < 0.001). There were no differences in other hemodynamic parameters between the two groups. Patients treated with esmolol required longer catecholamine support while no difference regarding survival, duration of invasive ventilation and ICU stay were noticed.

**Conclusion:**

No relevant hemodynamic difference was seen between neonates treated with perioperative esmolol and the control group and outcome did not differ. This indicates non-inferiority of perioperative betablocker therapy in young age. Prospective and placebo-controlled assessment of perioperative esmolol therapy in neonates is needed.

## Introduction

Dextro transposition of the great arteries (d-TGA) is one of the most common cyanotic congenital heart diseases and accounts for nearly 5% of all congenital heart defects [1]. Nowadays, Arterial Switch Operation (ASO) for correction of d-TGA is typically performed between the 5th and 14th day of life, and it is associated with excellent long-term outcome [2–4]. However, following ASO with cardio-pulmonary bypass (CPB), a significant number of infants develop pronounced capillary edema and restrictive dysfunction of the swollen myocardium. In those children, sinus tachycardia is frequently observed and often persists even after optimization of hemodynamics, such as preload optimization, afterload management and correction of acidosis. By shortening the diastolic filling period and thus the myocardial perfusion time, sinus tachycardia is thought to even deteriorate the diastolic dysfunction [5–7].

To address these postoperative challenges, interventions aimed at controlling heart rate are warranted. The short-acting beta-1-selective beta-blocker esmolol has shown promise in managing tachyardia-induced myocardial stress in various cardiac conditions. However, limited data are available regarding its efficacy and safety in neonatal cardiac surgery with cardiopulmonary bypass (CPB) for d-TGA [8–14].

Therefore, the objective of this study was to investigate the efficacy and safety of esmolol in controlling heart rate in neonatal cardiac surgery with CPB for d-TGA. By evaluating the impact of esmolol on heart rate, hemodynamics, laboratory findings and outcome in the distinct patient cohort of newborns after ASO for d-TGA, we aimed to contribute to the understanding of a heart rate orientated approach in the care for newborns following cardiac surgery within the first weeks of life.

## Material and Methods

For the purpose of this retrospective study, medical records of all neonates with d-TGA who underwent ASO within the first month of life between 01/2012 and 12/2021 at our institution were reviewed. Patients with need for postoperative extracorporeal life support were excluded from this study. During ASO, Custodiol® cardioplegic solution (30 ml/kg; Franz Köhler Chemie, Bensheim, Germany) was used in all children thorough the complete study period. The arterial switch operations were performed with mild hypothermia with a target temperature of 32 °C. Ultrafiltration was used during all operations. As a standard of care in our institution, postoperative heart rate is a cornerstone in the care of children following cardiac surgery with CBP. The use of epinephrine and dobutamine is avoided and inotropy is supported with Milrinone. Esmolol is part of this concept: postoperative use and dosage of esmolol to treat sinus tachycardia is at the individual discretion of the cardiac intensivist in charge with an aimed heart rate < 150/min. Afterload was adjusted with noradrenaline and vasopressin. The patient cohort was thus divided into individuals who received esmolol within the first 12 hours following CPB and those who did not. Baseline characteristics and laboratory findings were assessed as well as hemodynamics at the time of admission on the pediatric intensive care ward. A second hemodynamic and laboratory assessment was made in the early morning of the following day, after a medium time of 11 (IQR 9.5–11) hours following ICU admission. The number of days with noradrenaline support, invasive ventilation and stay in the pediatric ICU as well as survival > 30 days following CPB were assessed. Differences between groups were analyzed depending on data distribution: Mann–Whitney U-test for continuous variables and chi-square test for categorical variables. A *p*-value < 0.05 was considered significant.

We selected the lactate, hemoglobin, troponin I, arterial oxygen and central venous oxygen saturation from the standardized blood gas and blood samples taken at the different time points mentioned above.

## Results

A total of 112 subjects with ASO for d-TGA were included into this study of whom 57/112 (51%) children received esmolol within the first 12 h following CPB. Baseline characteristics of the study cohort are given in Table 1. The median age at surgery was 7 (IQR 5–9) days, median body weight was 3.4 (IQR3.0–3.8) kg, and median body length was 52 (IQR 49–54) cm. D-TGA was associated with ventricular septal defect in 26/112 (23%) subjects, 9/112 (8%) had additional coarctation of the aortic arch requiring arch repair while atypical coronary anatomy (including Cx from RCA) was present in 27/112 (24%) patients. Median CBP ischemic time was 115 (IQR 100–134) min. Perioperative junctional ectopic tachycardia (JET) occurred in 41/110 (37%) subjects. It is of note that there was no difference in those baseline data among patients treated with esmolol (*n* = 57) and the control group (*n* = 55). Esmolol dosing was started in 13/57 (23%) prior to ICU admission and in 44/57(77%) after a median time of 4.5 (IQR 1.3–10) hours following ICU admission. Esmolol dosage was titrated following heart rate with a median start dose of 31 (IQR 22–49) mcg/kg/min and a median maximum dose of 71 (46–101) mcg/kg/min.

**Table 1:** Baseline characteristics of the study cohort (*n* = 112)

	All Patients (*n* = 112)	Esmolol intervention group (*n* = 57)	Control group (*n* = 55)	*p*-value
Age at Surgery (days)	7 (5–9)	7 (5–9)	6 (5–8)	0.078
Body Weight (kg)	3.4 (3.0–3.8)	3.4 (3.0–3.8)	3.4 (3.2–3.7)	0.562
Body Length (cm)	52 (49–54)	51 (49–53)	52 (50–54)	0.202
Body Surface Area (m^2^)	0.21 (0.2–2.3)	0.21 (0.19–0.23)	0.21 (0.20–0.22)	0.337
Cardiac Bypass Ischemic Time (min)	115 (100–134)	112 (100–131)	116 (100–136)	0.746
VSD [*n* (%)]	26 (23%)	15 (26%)	11 (20%)	0.286
Aortic Arch Repair [*n* (%)]	9 (8%)	5 (9%)	4 (7%)	0.523
Atypical coronary anatomy [*n* (%)]	27 (24%)	16 (28%)	11 (20%)	0.219
Perioperative JET [*n* (%)]	41 (37%)	22 (39%)	19 (35%)	0.402
Chest closure				0.445
Primary chest closure [*n* (%)]	47 (42%)	24 (42%)	23 (42%)	
Temporary patch closure following surgery [*n* (%)]	51 (45%)	28 (49%)	23 (42%)	
Secondary chest decompression [*n* (%)]	14 (13%)	5 (9%)	9 (16%)	

Data are given as median and inter-quartile-range or number and percentage if appropriate

At ICU admission, the heart rate of subjects of the esmolol group was significantly higher (median heart rate 155 (IQR 150–163) min^−1^) when compared to the control group (147 (IQR 137–161) min^−1^; *p* = 0.018). There was no difference in any hemodynamic parameter at ICU admission between the groups, including central venous pressure and oxygen saturation, lactate and troponin concentration (see Table 2). At the time of second hemodynamic assessment (‘steady state hemodynamics’) heart rate was significantly lower in children treated with esmolol (median heart rate 135 (IQR 130–140) min^−1^ vs. 144 (IQR 150–163) min^−1^; *p* < 0.001). Body temperature as well as hemoglobin concentration was slightly lower among patients treated with esmolol though there was no other hemodynamic difference among the patient groups at this second assessment.

**Table 2:** Hemodynamic parameters after arterial switch operation

	All Patients (*n* = 112)	Esmolol intervention group (*n* = 57)	Control group (*n* = 55)	*p*-value
Heart Rate at ICU admission (bpm)	152 (143–162)	155 (150–163)	147 (137–161)	0.018*
Heart Rate at ‘steady state’ (bpm)	138 (131–149)	135 (130–140)	144 (150–163)	< 0.001*
At ICU admission:				
Body temperature (°C)	36.6 (36.2–36.9)	36.5 (36.2–37.0)	36.6 (36.4–36.9)	0.286
Blood Pressure – systolic (mmHg)	76 (69–85)	76 (68–83)	75 (71–86)	0.749
Blood Pressure – diastolic (mmHg)	50 (45–56)	52 (45–56)	49 (44–57)	0.600
Blood Pressure – amplitude (mmHg)	26 (22–30)	24 (22–30)	27 (23–31)	0.166
Central venous pressure (mmHg)	8 (7–10)	8 (7–10)	8 (6–10)	0.832
Arterial oxygen saturation (%)	99.6 (99.0–100)	99.5 (98.9–100)	99.8 (99.2–100)	0.085
Central venous oxygen saturation (%)	62.8 (51.8–73.1)	64 (53–74)	63 (51–73)	0.682
AVDO_2_ (%)	36.3 (26.8–47.7)	35.8 (26.5–46.3)	37.2 (26.9–47.8)	0.609
NIRS (%)	58 (50–63)	58 (49–62)	59 (50–66)	0.224
Lactate concentration (mmol/dl)	1.8 (1.5–2.3)	1.7 (1.4–2.4)	1.8 (1.5–2.2)	0.694
Troponin I concentration (µg/l)	37.4 (26.9–56.9)	38.4 (24.6–57.7)	37.1 (28.8–54.1)	0.816
Hemoglobin concentration (g/dl)	12.5 (11.2–13.9)	12.5 (11.5–14.3)	12.4 (10.8–13.5)	0.146
At steady state’ hemodynamics:				
Body temperature (°C)	35.7 (35.3–36.1)	35.4 (34.9–36.0)	35.9 (35.5–36.3)	0.001*
Blood Pressure – systolic (mmHg)	65 (60–71)	65 (60–72)	63 (59–71)	0.416
Blood Pressure – diastolic (mmHg)	44 (41–48)	45 (41–48)	44 (41–50)	0.9357
Blood Pressure – amplitude (mmHg)	21 (17–25)	21 (17–25)	20 (18–24)	0.395
Central venous pressure (mmHg)	8 (6–9)	8 (6–10)	7 (6–9)	0.231
Arterial oxygen saturation (%)	98.5 (97.4–99.2)	98.4 (97.6–99.2)	98.5 (97.4–99.1)	0.967
Central venous oxygen saturation (%)	69.2 (58.5–75.2)	69 (59–74)	69 (57–75)	0.896
AVDO_2_ (%)	29.9 (22.0–38.0)	30.1 (22.8–36.0)	29.0 (20.7–39.6)	0.782
NIRS (%)	66 (60–74)	65 (59–74)	66 (61–73)	0.968
Lactate concentration (mmol/dl)	2.1 (1.7–2.7)	2.2 (1.8–2.7)	2.1 (1.7–2.5)	0.379
Troponin I concentration (µg/l)	24.5 (17.7–38.2)	25.5 (18.2.40.9)	22.8 (15.5–33.6)	0.098
Hemoglobin concentration (g/dl)	14.3 (13.0–15.6)	13.9 (12.4–15.0)	14.6 (13.3–15.9)	0.024*

Data are given as Medina and interquartile range, while differences between the groups are calculated with U-test

All subjects survived > 30 days following ASO (see Table 3). Noradrenalin support was needed for a median duration of 3 (IQR 2–5) days following ASO, with subjects of the esmolol group having a significantly longer need for support compared to the control group (*p* = 0.01). There was no significant difference observed in the duration of Milrinone support, duration of invasive ventilation, and total number of days spent on the ICU between the esmolol and control groups.

**Table 3:** Outcome after ASO

	All Patients (*n* = 112)	Esmolol intervention group (*n* = 57)	Control group (*n* = 55)	*p*-value
30 days survival [*n* (%)]	112 (100%)	57 (100%)	55 (100%)	n/a
Days with inotropic support [*n* (IQR)]	3 (2–5)	4 (2–5)	3 (2–4)	0.011*
Days with Milrinone support [*n* (IQR)]	5 (3–6)	5 (4–7)	4 (3–5)	0.072
Days with invasive ventilation [*n* (IQR)]	4 (3–6)	4 (3–6)	4 (2–6)	0.514
Days on ICU [*n* (IQR)]	8 (6–11)	8 (6–10)	8 (6–11)	0.974

Data are given as Median and interquartile range, while differences between the groups are calculated with U-test

## Discussion

The objective of this study was to investigate the efficacy and safety of esmolol in controlling heart rate in neonatal cardiac surgery with CPB. We focused on subjects with d-TGA following ASO as they form a relative homogenous group among the vast variety of neonatal cardiac surgery. The findings of this cohort could therefore be carefully extrapolated as a model of neonatal cardiac surgery.

Sinus tachycardia is a common problem after cardiac surgery at a very young age and can potentially impact cardiac function by reducing diastolic filling time and myocardial perfusion while increasing myocardial oxygen consumption. Postoperative myocardial edema most severely affects the diastolic function in those young hearts as the relative volume loss of neonatal heart chambers due to swelling is significant. To maintain cardiac output, the neonatal heart develops sinus tachycardia which decreases the diastolic pressure–time integral, which is the driving force for coronary blood flow and of utmost importance after transfer of the coronary arteries as a part of ASO. Due to this assumption, a heart rate orientated postoperative intensive care was performed with esmolol used to treat sinus tachycardia. It is important to state that other measures affecting heart rate like active cooling or arrhythmia management were identical between children treated with or without esmolol. We did observe, however, no relevant difference in the hemodynamic parameters other than heart rate both at ICU admission and after 12 hours between individuals treated with esmolol and the control group. Importantly, lactate, central venous SO_2_ or troponin I concentrations were equal between the two groups indicating that lowering the heart rate did not induce an oxygen debt in the postoperative circulation.

It is of note that physicians were not blinded for the use of esmolol: It must be assumed that children treated with esmolol were deemed to have a more pronounced edema than those of the control group. Hemodynamic equality during the immediate postoperative course between children treated with and without esmolol could therefore indicate the efficacy of the postoperative heart rate reduction. In line with this assumption, children treated with esmolol required longer ionotropic support, most properly due to the side effects of the betablocker. However, survival and duration of ICU stay were not different compared to the control group which underlines the safety of the heart rate orientated postoperative intensive care.

In an irany study, esmolol was used during CPB in 30 children undergoing closure of atrial and ventricular septal defects and were compared to a control group. In this relatively small retrospective analysis, children treated with esmolol had a higher LV-EF one week following surgery while no effects on ventilation time or ICU stay were noticed [10]. Other retrospective single center reports on children after repair of Tetralogy of Fallot [12] or VSD closure [13] reported no adverse effects on a systematic perioperative esmolol dosage. Those children were older than our cohort and the benefit of perioperative heart rate reduction would theoretically be smaller in that age due to the reasons listed above. No benefits of perioperative betablockers though are described for adults following cardiac surgery with CPB [13, 14]. It has to be stated that several limitations that are founded by the retrospective design of this study (like lack of systematic echocardiography and volume administration) make interpretation difficult.

To prove the efficacy of that heart rate oriented postoperative care in neonates independently, a prospective placebo-controlled analysis which avoids these limitations is mandatory and will be conducted soon.

## Data Availability

No datasets were generated or analysed during the current study.
